# *Nocardia brasiliensis* Infection Complicating Cryptogenic Organizing Pneumonia

**DOI:** 10.1155/2017/9567175

**Published:** 2017-02-28

**Authors:** Alison M. Fernandes, Jason C. Sluzevich, Isabel Mira-Avendano

**Affiliations:** ^1^Saba University School of Medicine, The Bottom, Saba, Netherlands; ^2^Department of Dermatology, Mayo Clinic, Jacksonville, FL, USA; ^3^Division of Pulmonary, Allergy and Sleep Medicine, Mayo Clinic, Jacksonville, FL, USA

## Abstract

Pulmonary nocardiosis is a severe and uncommon opportunistic infection caused by* Nocardia* species. We present a patient with cryptogenic organizing pneumonia who was receiving long-term immunosuppressive therapy, whose treatment course was complicated by cutaneous and pulmonary nocardiosis. Tissue cultures confirmed* Nocardia brasiliensis*. Nocardiosis should be a diagnostic consideration for patients treated with long-term immunosuppression who have worsening pulmonary symptoms and relapsing pustular skin lesions.

## 1. Introduction

Cryptogenic organizing pneumonia (COP) is both a fibrotic and an inflammatory change in the lung where the inciting injury is rarely recognized. Its clinical presentation mimics community-acquired pneumonia and includes such symptoms as progressive dyspnea, nonproductive cough, and a flu-like prodrome [1]. If left untreated, COP outcomes can range from spontaneous remission to death [1]. The mainstay of therapy for COP is high-dose corticosteroids resulting in resolution in 70% to 80% of all cases [2]. While clinical improvement can be seen within 48 hours of starting oral corticosteroids, longer treatment durations are often necessary to fully clear pulmonary infiltrates and prevent relapse [1]. The most commonly reported corticosteroid-related adverse events with chronic corticosteroid therapy include Cushing syndrome, adrenal insufficiency, steroid-induced myopathy, and diabetes mellitus. In addition, systemic corticosteroids, especially when combined with other antimetabolite immunosuppressives, may increases the risk of opportunistic infection.

## 2. Case Report

A 71 y/o man with an environmental exposure to insulation for 20 years while working at NASA, with less than a 10-pack year history of smoking presented to the community emergency department with severe dyspnea and low grade fever. He was diagnosed and admitted for bacterial pneumonia, treated for two days with IV antibiotics, and discharged on oral antibiotics. His symptoms did not improve prompting two additional hospital admissions over 3 months. Chest CT ultimately demonstrated intraseptal thickening, areas of peribronchial consolidation, and ground-glass opacity involving the lower lobes. A surgical lung biopsy revealed organizing pneumonia associated with alveolar septal inflammation leading to a final diagnosis of COP. The patient was initially treated with Prednisone 80 mg daily that was later tapered to 60 mg 4 weeks later. Azathioprine was also started at 100 mg daily as a steroid sparing agent. Despite these interventions, his dyspnea continued and he was referred to our institution for further evaluation. On presentation, several painful, variably pustular papules and small nodules were present on the face and extremities. He reported an outdoor fall 8 weeks earlier and unsuccessful treatment with both doxycycline and cephalexin given for one month.

**Figure 1:**
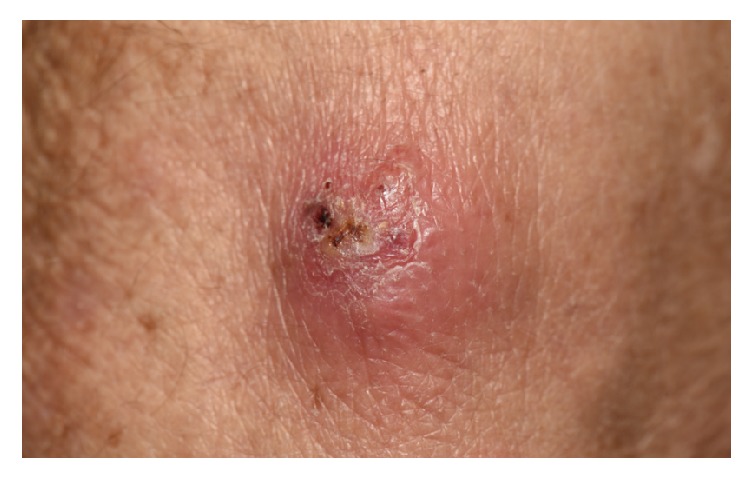
Lesional skin. Fluctuant and purulent-crusted nodule.

**Figure 2:**
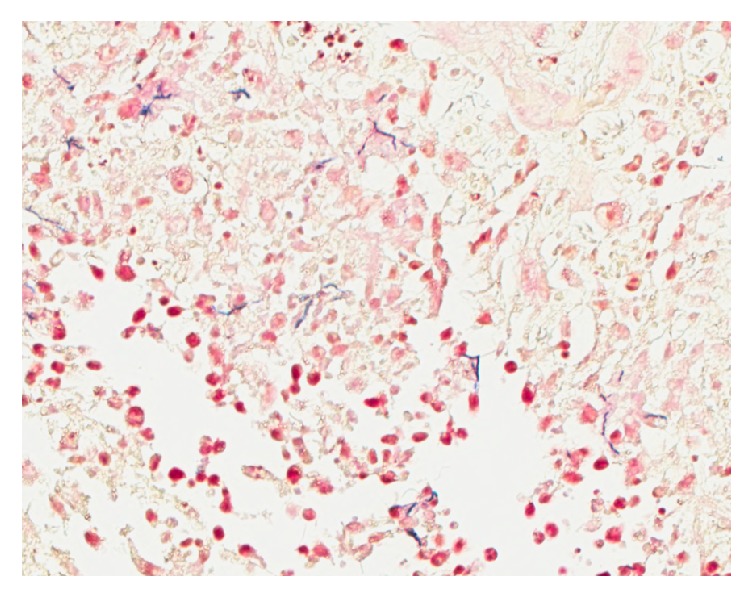
Filamentous gram-positive rods. Gram stain, original magnification ×40.

A biopsy by 4 mm punch technique of a fluctuant nodule on the arm (Figure 1) revealed a dermal abscess with gram-positive filamentous rods characteristic of* Nocardia* species (Figure 2). A modified acid-fast Kinyoun stain was also positive. Chest CT showed interval development of bilateral upper lung nodules. The patient was directly admitted to the hospital and began treatment with intravenous imipenem in conjunction with oral trimethoprim-sulfamethoxazole (TMP-SMX). Magnetic resonance imaging of the brain and a CT scan of the abdomen and pelvis were negative for other end-organ involvement. Tissue cultures grew* Nocardia brasiliensis* susceptible to augmentin and TMP-SMX.

The patient was discharged 5 days later after showing clinical improvement, with plans to continue TMP-SMX therapy for 6 months. At follow-up 5 weeks after discharge, all cutaneous lesions had cleared, and repeat chest CT showed remarkable improvement with only minimal left upper lobe involvement. The Pulmonary Function Test completed reported minimal airway resistance and negligible airway obstruction.

## 3. Discussion

Organizing pneumonia is defined pathologically by the presence of buds of granulation tissue in the distal air spaces. The lesions occur predominantly within the alveolar areas, but sometimes there is associated compromise of the bronchial lumen. The pathological pattern is not specific for any disorder or cause but reflects an inflammatory process secondary to lung injury; however, it is the particular hallmark of a recognized clinic-radiological entity known as an cryptogenic organizing pneumonia, when etiology cannot be established [3, 4]. This entity is characterized by good response to steroid therapy, but some cases especially those with reticulation on high resolution CT or with fibrosing variant pathologic type can be refractory to this therapy. In these cases, steroid sparing immunosuppressive therapy using cyclosporine or azathioprine may be utilized, but the benefit of this has been not clearly established [5, 6].

As a potential opportunistic pathogen,* Nocardia* species is prevalent in populations that have compromised cell-mediated immunity. The immunocompetence of the host determines the rate and course of infection [7]. High-dose prolonged corticosteroid therapy is a known independent risk factor for infection with* Nocardia* because it suppresses T_H_1 cellular immunity [7, 8]. Most cases of disseminated or pulmonary nocardiosis are associated with* Nocardia asteroides *and have also been previously described to be associated with COP. Structural alternations seen with chronic pulmonary disease, such as COP in this case, increase the likelihood of pulmonary colonization by* Nocardia* and can present as acute, subacute, or chronic pneumonia [7].

In this case, the patient acquired nocardiosis most likely through direct cutaneous inoculation, given a facial laceration after a fall outdoors and a tissue culture showing* N. brasiliensis*, which is the most common cause of cutaneous nocardiosis. As these lesions developed after treatment for progressive dyspnea, we postulate the degree of iatrogenic immunosuppression was permissive for hematogenous dissemination of the* Nocardia* to the lung. While the respiratory tract is typically the main entry point for these organisms, dissemination of* Nocardia* can occur from either a pulmonary or a cutaneous source to any organ [9]. In immunocompetent hosts, primary cutaneous nocardiosis rarely disseminates and generally presents with localized pustules or as a small abscess that may ulcerate. In immunocompromised hosts, hematogenous spread to lung, liver, and central nervous system is a possible complication that can be fatal [7] and multiple relapsing cutaneous pustular lesions are commonly observed.

*Nocardia* does not belong to normal human flora and is seldom a contaminant in tissue culture [7]. It is found in soil, decaying plants, and dust particles [10]. Cultures are slow to grow and are at risk of being overgrown by bacterial containments, thereby leading to false-negative findings [11]. Gram stain has the highest sensitivity for detection of* Nocardia* in tissue [12]. The bacterium appears as gram-positive, beaded, branching, filamentous rods that are uniquely and weakly acid-fast positive. This staining combination can suggest* Nocardia* as a possible cause even before speciation by culture is available. Use of modified acid-fast stains, such as Kinyoun stain, also improves visualization [12].

Sulfonamides are frequently a component in the empirical treatment of nocardiosis [13]. However, sulfonamide monotherapy has been associated with increased mortality rate in some reports, and therefore combination therapy should be considered for extensive and disseminated cases [7]. One recommended approach is an initial regimen of TMP/SMX in combination with a fluoroquinolone, but alternative treatments also use minocycline, imipenem, linezolid, third-generation cephalosporins, dapsone, erythromycin, clindamycin, and amikacin [7, 8, 11, 13], either alone or in combination. Final therapy ultimately should be tailored to the sensitivity panel from culture. In current literature, consensus on duration and dosage is poor, but the immune status of the patient is likely important [7]. Previous case studies have suggested 3-month treatment for immunocompetent patients and 6 months for immunocompromised patients [12].

The prognosis of localized nocardiosis is favorable; however, disseminated nocardiosis can be fatal, especially with central nervous system involvement [12]. Therefore, consideration of nocardiosis as a diagnostic factor is important for immunocompromised patients, especially those with preexisting pulmonary disease and a history of relapsing skin lesions. Treating physicians should be aware that the failure of corticosteroids to improve COP, especially in the setting of other immunosuppressive medications, may reflect a secondary iatrogenic infection requiring specific antibiotic therapy.

## Abbreviations

COP: Cryptogenic organizing pneumonia
CT: Computed tomography
TMP-SMX: Trimethoprim-sulfamethoxazole.
